# Comparison and verification of turbulence Reynolds-averaged Navier–Stokes closures to model spatially varied flows

**DOI:** 10.1038/s41598-020-76128-9

**Published:** 2020-11-04

**Authors:** Kudzai Chipongo, Mehdi Khiadani, Kaveh Sookhak Lari

**Affiliations:** 1 School of Engineering, Edith Cowan University, 270 Joondalup Drive, Joondalup, WA 6027 Australia; 2 CSIRO Land and Water, Private Bag No. 5, Wembley, WA 6913 Australia

**Keywords:** Civil engineering, Environmental impact, Limnology

## Abstract

The robustness and accuracy of Reynolds-averaged Navier–Stokes (RANS) models was investigated for complex turbulent flow in an open channel receiving lateral inflow, also known as spatially varied flow with increasing discharge (SVF). The three RANS turbulence models tested include realizable *k*–*ε*, shear stress transport *k*–*ω* and Reynolds stress model based on their prominence to model jets in crossflows. Results were compared to experimental laser Doppler velocimetry measurements from a previous study. RANS results in the uniform flow region and farther from the jet centreline were more accurate than within the lateral inflow region. On the leeward side of the jet, RANS models failed to capture the downward velocity vectors resulting in major deviations in vertical velocity. Among RANS models minor variations were noted at impingement and near the water surface. Regardless of inadequately predicting complex characteristics of SVF, RANS models matched experimental water surface profiles and proved more superior to the theoretical approach currently used for design purposes.

## Introduction

The interaction of lateral inflow with a co-stream in an open channel generates strong turbulence due to flow mixing, air entrainment and the possible formation of vortices^1^. Lateral inflow discharging into side and collector channels is a common example of a spatially varied flow (SVF) with a wide range of applications^2,3^. These include roof gutters, wash water gutters at treatment plants, road ditches, drainage conduits, side channel spillways as well as the more complex unsteady overland flow due to rainfall^2,4–10^.

Current models are based on a number of assumptions, including that air entrainment is negligible, velocity distribution is uniform and that resistance can be estimated using equations for uniform open channel flow. Hager and Bremen^11^ and Lucas et al.^1^ described the flow conditions associated with the formation of single and twin vortices with air entrainment and the effects on the transverse water surface profile. Many researchers have also reported that the Manning, Chezy and Darcy–Weisbach equations underestimate flow resistance in SVF^2,4,5,12,13^. However, methods for predicting more representative resistance factors are not yet available^14,15^. The effect of non-uniform velocity distributions is often incorporated in a single momentum correction factor, *β*^1,3^. As *β* cannot be easily quantified, such an approach is often perceived as an over-complication^1,3^. Yet, its exclusion leads to inconsistent water surface profiles for some applications of SVF. Nonetheless, the simplifications permit one-dimensional approach to estimate the stream-wise water surface profile^16–21^.

A few studies for the turbulence characteristics of SVF also exist. Yoon and Wenzel^22^ measured longitudinal and Kisisel^23^ both longitudinal and vertical turbulence intensities respectively. The former concluded that turbulence intensities decreased with increasing Reynolds number towards the channel bed, while the latter reported strong turbulence intensities near the surface. Khiadani^24^ applied laser Doppler velocimetry (LDV) measurements in a controlled environment with a lateral inflow discharged very close to the free-surface and minimum air entrainment. The author concluded that SVF velocity and turbulence distributions were too complex, particularly within the inflow region, to develop empirical equations similar to uniform flow^25^ and that more complex models were required to identify flow structures and turbulence characteristics^7^. Nezu^26^ comprehensively reviewed turbulence modelling studies on various types of open channel flow. Stoesser^27^ discussed the challenges of applying Large Eddy Simulation (LES) and Direct Numerical Simulation (DNS) in hydraulics, namely the requirement of a super-refined grid and computing power restrictions for the complex flow physics at high Reynolds numbers. Reynolds-Averaged Navier–Stokes (RANS) resolves average quantities and models all turbulent fluctuations. It has gained widespread use mainly due to its low computing power requirements and relative success in many applications, including jets in crossflows^28–31^.

This study attempts to replicate the experimental turbulence results of Khiadani^24^ using RANS models and comments on the relevance of their application for predicting turbulence characteristics in SVF. Such results could then be used to generate more data to be used to develop empirical equations for estimating turbulence characteristics of open channel flows with lateral discharge.

### Turbulence models

Turbulent flows comprise random temporal and spatial fluctuations. The presence of rough boundaries, obstacles or strong mixing introduce high disturbance in the flow. Reynolds-averaged Navier–Stokes (RANS) equations, which are derived from time averaging Navier–Stokes equations are often utilised to simulate turbulent flows in industrial applications. RANS models employ an empirical closure hypotheses to compute the components of the Reynolds stress tensor^32^. Classification of RANS models is based on the number of additional differential transport equations required to determine turbulence quantities. 2-equation closures (also referred to as first order moment closures) solve two transport equations, one for turbulent kinetic energy, *k* and an additional equation for the rate of dissipation, *ε* or the specific dissipation rate, *ω*^33,34^. Reynolds stresses are related to the instantaneous mean rate of strain tensor using the following Boussinesq eddy viscosity assumption:

1 $$ - \overline{{u_{i} u_{j} }} = \nu_{t} \left( {\frac{{\partial U_{i} }}{{\partial x_{j} }} + \frac{{\partial U_{j} }}{{\partial x_{i} }}} \right) - \frac{2}{3}\delta_{ij} k $$

where ${\nu }_{t}={\mu }_{t}/\rho$= kinematic turbulent eddy viscosity; $k = 1/2\left( {\overline{{u^{\prime 2} }} + \overline{{v^{\prime 2} }} + \overline{{w^{\prime 2} }} } \right)$ = turbulent kinetic energy, ${\delta }_{ij}$ = Kronecker delta and is equal to 1 and 0 if *i* = *j* and *i* ≠ *j*, respectively. Strain is the primary medium through which turbulence is generated and sustained and thus plays a pivotal role in the closure process. 2-equation models can therefore predict Reynolds stresses and isotropic turbulence without solving extra equations. This results in simpler models and computational savings in time and effort. However, this simplification results in the limitations of the eddy viscosity models. Reynolds stress is assumed proportional to the mean flow strain tensor. This is valid in simple flows e.g. straight boundaries and wakes but is invalid in intricate flows with streamline curvature^35–37^, separation^33,38^, rapid acceleration and deceleration (or stagnation)^39^. In addition, since the instantaneous value of mean strain rate is used flow evolution history is disregarded^40^.

Reynolds stress models (RSM), or second order moment closures are more complex. An additional six differential equations are solved to describe Reynolds stresses and all mean flow properties. The extra transport equations include terms for production, dissipation, diffusion, turbulent pressure-strain interactions and rotations. It is often presumed that RSM which solves Reynolds stresses using transport equations to predict anisotropy of Reynolds stresses is superior over two-equation models which use an isotropic eddy viscosity approach^29,34^.

Turbulence closures applied here include the 2-equation models, *k*–*ε*, *k*–*ω* and Reynolds Stress model (RSM)^41,42^. Although the *k*–*ε* is by far the most extensively validated and used, tests and applications of the *k*–*ε* have been successful for a wide range of flow conditions but inadequate in some^26,34,43–49^. Standard and RNG *k*–*ε* are appropriate for high speed and swirl flows^34,50^ while the realizable *k*–*ε* has considerable edge for modelling flows with important streamline curvature effects, vortices, and rotation such as near-wall modelling in impinging jet flows^51,52^ and flow after a backward step^53^. Jets in crossflow studies^54–56^ found realizable *k*–*ε* model predicts results that match experimental data. Menter^42^ proposed shear stress transport (SST) *k*–*ω* models by combining strengths of both standard *k*–*ω* and *k*–*ε* turbulence models resulting in the following distinct features; a gradual change from the standard *k*–*ω* model to the *k*–*ε* model in the inner to the outer region of the boundary layer, respectively using blending functions, and a modified turbulent viscosity equation for accurately capturing the transport effects for the principal turbulent shear stresses.

The Gibson and Launder^57^ pressure-strain Reynolds stress model has been well tested in many cases for impinging jets in a cross flow^29,30,58^. From the dissipation rate of turbulent kinetic energy, RSM can compute the destruction of turbulence as well as the anisotropic behaviour of Reynolds stresses unlike models that employ the Boussinesq approximation^59^. However, in some instances the additional computational costs undermine the benefits. Ostheimer and Yang^29^ mentioned that the RSM required approximately 3 times more CPU time compared to the *k*–*ε* but reported no significant difference in mean flow properties for a case of twin impinging jets in a crossflow. The Gibson and Launder^57^ pressure strain model was used in the current study. Transport closure for scalar turbulent diffusivity were estimated according to Lien and Leschziner^60^, Fu et al.^61^ and the pressure strain term as detailed by Gibson and Launder^57^ and Launder^62,63^. Enhanced wall treatment was applied and to avoid numerical instabilities, modified wall effect constants were defined as functions of Reynolds stress invariants and the turbulent Reynolds number as suggested by Launder and Shima^64^.

In plunging and impinging jets in crossflow the realizable *k*–*ε*, SST *k*–*ω* and the RSM yields results that better match experiments compared to other turbulence models employing RANS closures^52^. These models were applied in this study and their development is summarized in this section; the reader is referred to Hanjalić and Launder^65^, Hanjalić and Launder^65^, Launder and Sandham^66^, Leschziner^67^, Tu et al.^68^ and Versteeg and Malalasekera^34^, for detailed information on RANS models. The selected turbulence models solve the following generic equation:

2 $$ \rho \frac{{\partial \overline{\varphi }}}{\partial t} + \rho \overline{u}_{j} \frac{{\partial \overline{\varphi }}}{{\partial x_{j} }} - \frac{\partial }{{\partial x_{j} }}\left[ {\Gamma_{\varphi ,eff} \frac{{\partial \overline{\varphi }}}{{\partial x_{j} }}} \right] = S_{\varphi } $$

where *ϕ* represents variables, $\Gamma_{\varphi ,eff}$ represents the effective diffusion coefficient, and $S_{\varphi }$ represents the source term of an equation (see Table 1 for full mathematical expressions).

**Table 1:** Summary of selected turbulence closures and coefficients.

	Realizable *k*–*ε*^88^	SST *k*–*ω*^42^	RSM^57^
Variables, *ϕ*	*k*, *ε*	*k*, *ω*	$\overline{{u^{\prime}_{i} u^{\prime}_{j} }}$
Effective diffusion coefficient, $\Gamma_{\varphi ,eff}$	$\mu + \frac{{u_{t} }}{{\sigma_{k,t} }}$ $\mu + \frac{{u_{t} }}{{\sigma_{\varepsilon ,t} }}$	$\mu + \frac{{u_{t} }}{{\sigma_{k} }}$ $\mu + \frac{{u_{t} }}{{\sigma_{\omega } }}$	$\mu + \frac{{u_{t} }}{{\sigma_{ij} }}$
Source term, *S*_*ϕ*_	$G_{k} - \rho \varepsilon + G_{B}$ $C_{\varepsilon ,1} G_{k} \frac{\varepsilon }{k} - C_{\varepsilon ,2} \rho \frac{{\varepsilon^{2} }}{k}$	$\tilde{G}_{k} - Y_{k}$ $G_{\omega } - Y_{\omega } + D_{\omega }$	$P_{ij} + G_{ij} + \varphi_{ij} + \varepsilon_{ij}$
Constants and coefficients	$$\mu_{t} = \rho C_{\mu } \frac{{k^{2} }}{\varepsilon }$$ $$C_{\mu } = \frac{1}{{A_{0} + A_{s} \left( {\frac{{U^{*} }}{\varepsilon }} \right)}}$$ $$U^{*} = \sqrt {S_{ij} S_{ij} + \tilde{\Omega }_{ij} \tilde{\Omega }_{ij} }$$ $$\tilde{\Omega }_{ij} = \Omega_{ij} - 2\varepsilon_{ijk} \omega_{k}$$ $$\Omega_{ij} = \overline{{\Omega_{ij} }} - \varepsilon_{ijk} \omega_{k}$$ $$A_{s} = \sqrt 6 \cos \phi$$ $$\varphi = \frac{1}{3}\cos^{ - 1} \left( {\sqrt 6 W} \right)$$ $$W = \frac{{S_{ij} S_{jk} S_{ki} }}{{\sqrt {S_{ij} S_{ij} } }}$$ $$G_{k} = \mu_{t} S_{ij} S_{ij}$$ $$G_{B} = \beta g_{i} \left( {\frac{{\mu_{t} }}{{\sigma_{T,t} }}} \right)\frac{\partial T}{{\partial x}}$$ *A*_0_ = 4.04, *C*_*ε1*_ = 1.44, *C*_*ε2*_ = 1.92, *σ*_*k*_ = 1.00, *σ*_*ε*_ = 1.30, *σ*_*T,t*_ = 0.90, *σ*_*C,t*_ = 1.0	$$\mu_{t} = \frac{\rho k}{\omega }\frac{1}{{\max \left[ {\frac{1}{{\alpha^{*} }},\frac{{F_{2} \sqrt {S_{ij} S_{ij} } }}{{a_{1} \omega }}} \right]}}$$ $$\alpha^{*} = \frac{{\frac{{\beta_{i} }}{3} + \left( {\frac{{{\text{Re}}_{t} }}{6}} \right)}}{{1 + \left( {\frac{{{\text{Re}}_{t} }}{6}} \right)}}$$ $${\text{Re}}_{t} = \frac{\rho k}{{\mu \omega }}$$ $$\beta^{*} = \beta_{\infty }^{*} \frac{{\frac{4}{5} + \left( {\frac{{{\text{Re}}_{t} }}{8}} \right)^{4} }}{{1 + \left( {{\text{Re}}_{t} + 8} \right)^{4} }}$$ $$\tilde{G}_{k} = \min \left( {G_{k} ,10\rho \beta^{*} k\omega } \right)$$ $$G_{k} = 2\mu_{t} S_{ij} S_{ij} - \frac{2}{3}\rho k\frac{{\partial u_{i} }}{{\partial x_{j} }}\delta_{ij}$$ $$Y_{k} = \rho \beta^{*} k\omega$$ $$Y_{\omega } = \rho \beta \omega^{2}$$ $$G_{\omega } = \frac{{\rho \alpha G_{k} }}{{\mu_{t} }}$$ $$D_{\omega } = 2\left( {1 - F_{1} } \right)\rho \sigma_{\omega ,2} \frac{1}{\omega }\frac{\partial k}{{\partial x_{j} }}\frac{\partial \omega }{{\partial x_{j} }}$$ *σ*_*k,1*_ = 1.176, *σ*_*k,2*_ = 1.0, *σ*_*ω,1*_ = 2.0, *σ*_*ω,2*_ = 1.168_*,*_* a*_1_ = 0.31	$$P_{ij} = - \rho \left( {\overline{{u^{\prime}_{i} u^{\prime}_{k} }} \frac{{\partial u_{j} }}{{\partial x_{m} }} + \overline{{u^{\prime}_{j} u^{\prime}_{k} }} \frac{{\partial u_{i} }}{{\partial x_{m} }}} \right)$$$$G_{ij} = - \rho \beta \left( {g_{i} \overline{{u^{\prime}_{j} T}} + g_{j} \overline{{u^{\prime}_{i} T}} } \right)$$ $$\varphi_{ij} = \overline{{p\left( {\frac{{\partial u^{\prime}_{i} }}{{\partial x_{j} }} + \frac{{\partial u^{\prime}_{j} }}{{\partial x_{i} }}} \right)}}$$ $$\varepsilon_{ij} = \frac{2}{3}\delta_{ij} \varepsilon$$; *σ*_*ε,1*_ = 1.0, *C*_*ε,1*_ = 1.44. *C*_*ε,2*_ = 1.92.

*u*_*i* _= velocity component in *i*-direction, *T* = temperature, *k* = kinetic energy of turbulence, *ε* = dissipation rate of turbulent kinetic energy, *ω* = specific dissipation rate of *k*, *p* = pressure, *μ*_*t*_ = eddy viscosity, *G*_*ϕ*_ = turbulence production for *ϕ*, and *S* = rate of the strain, *G*_*B* _= buoyancy production term, *F* = weighting function in SST *k*–*ω*, *G*_*k*_ = diffusion coefficient, *i*, *j*, *m* = Cartesian coefficients, Re = Reynolds number, ρ = density of flow, $\overline{{\Omega_{ij} }}$ = mean rate of rotation tensor in a frame moving with an angular velocity *ω*_*k*_, *σ*_*k*_ and *σ*_*ω*_ = turbulent Prandtl number, and α^*^ = factor used to damp the turbulent viscosity resulting in a low-Reynolds number correction.

## Materials and methods

### Experimental validation of numerical models

The experimental data of Khiadani^24^ was used to validate current numerical models. Numerical models were developed using the experimental results as boundary conditions where possible. Experiments were conducted in a 7.5 m long, 0.4 m wide channel with 0.2 m side-walls. Lateral inflow was provided by 16 circular nozzles of diameter 28 mm, 123 mm apart (centre to centre). The centre of the first nozzle was at *x* = 4.702 m. All nozzles were discharging perpendicular to the channel bed, at a constant height of 80 mm and the slope of the channel, *S*_0_ was set to 0.3%. A base flow of 5 L s^−1^ was provided and two lateral inflow rates were studied; *q* = 2.14 and 2.82 L s^−1^ m^−1^. Flow conditions for the experimental conditions of Khiadani^24^ are summarized in Table 2. Measurements of average flow depth were taken at 0.1 m intervals within the lateral inflow region, as well as one location each in the uniform flow region upstream and downstream of the inflow zone (see Table 2) using a depth gauge with an accuracy of ± 0.05 mm. Due to surface waves at the channel centreline at which inflow was discharging, measurements were taken approximately 75 mm away from the channel centreline. Variations in water depth across the width of the channel were insignificant.

**Table 2:** Experimental conditions for *q* = 2.14 L s^−1^ m^−1^^24^.

*N*	*x* (m)	*h*(*x*) (m)	*A* (m^2^)	*R*_*h*_ (m)	*Q* (m^3^ s^−1^)	*U* (m s^−1^)	*v*_*j*_ (m s^−1^)	*r* = *v*_*j*_/*U* (−)	Re (−)	F (−)
*	4.56	6.03 × 10^–2^	2.41 × 10^–2^	4.63 × 10^–2^	0.50 × 10^–2^	2.07 × 10^–1^	0.41	1.96	3.83 × 10^4^	0.27
1	4.76	6.04 × 10^–2^	2.41 × 10^–2^	4.64 × 10^–2^	0.52 × 10^–2^	2.14 × 10^–1^	0.41	1.90	3.96 × 10^4^	0.28
3	5.00	6.02 × 10^–2^	2.41 × 10^–2^	4.62 × 10^–2^	0.57 × 10^–2^	2.36 × 10^–1^	0.41	1.72	4.36 × 10^4^	0.31
5	5.26	5.92 × 10^–2^	2.37 × 10^–2^	4.56 × 10^–2^	0.62 × 10^–2^	2.63 × 10^–1^	0.41	1.55	4.78 × 10^4^	0.34
7	5.50	5.78 × 10^–2^	2.31 × 10^–2^	4.48 × 10^–2^	0.67 × 10^–2^	2.92 × 10^–1^	0.41	1.39	5.22 × 10^4^	0.39
9	5.75	5.67 × 10^–2^	2.27 × 10^–2^	4.42 × 10^–2^	0.73 × 10^–2^	3.20 × 10^–1^	0.41	1.27	5.65 × 10^4^	0.43
11	5.99	5.39 × 10^–2^	2.16 × 10^–2^	4.24 × 10^–2^	0.78 × 10^–2^	3.61 × 10^–1^	0.41	1.12	6.12 × 10^4^	0.50
13	6.24	5.18 × 10^–2^	2.07 × 10^–2^	4.11 × 10^–2^	0.83 × 10^–2^	4.01 × 10^–1^	0.41	1.01	6.59 × 10^4^	0.56
15	6.49	4.33 × 10^–2^	1.73 × 10^–2^	3.56 × 10^–2^	0.88 × 10^–2^	5.10 × 10^–1^	0.41	0.80	7.25 × 10^4^	0.78
#	7.05	4.25 × 10^–2^	1.70 × 10^–2^	3.51 × 10^–2^	0.90 × 10^–2^	5.29 × 10^–1^	0.41	0.77	7.41 × 10^4^	0.82

*Upstream of the inflow zone.

^#^Downstream of the inflow zone.

LDV measurements of point velocities and fluctuations were obtained within a three-dimensional grid. In the longitudinal direction and within the lateral inflow region, measurements were taken half-way between the centres of two consecutive nozzles plus one location each in the uniform flow region upstream and downstream. In the span wise direction, due to symmetry, measurements were taken at *z* = 0, 0.075 and 0.15 m where *z* = 0 is at the centre of the channel. For detailed flow measurements between two jets at the channel centreline in the longitudinal direction, measurements were conducted from half-way between the 7th and 8th jets to half way between the 9th and 10th jets at 20 mm intervals. Thus 13 measurements were taken from *x* = 5.5015 mm to 5.7475 m. Likewise for detailed measurements in the span wise direction at *x* = 5.0095, 5.5015, and 5.9935 m measurements were made at *z* = 0, 0.025, 0.050, 0.075, 0.100, 0.125, 0.150, and 0.175 m. Along the depth, an appropriate number of measurements were taken from the bed to the free-surface.

### Computational domain

**Figure 1:**
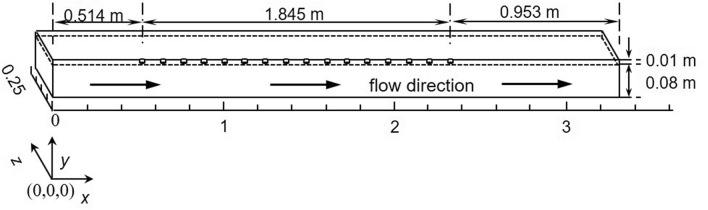
Computational geometry.

The geometry of the numerical solution is illustrated in Fig. 1; the origin is considered to be at the bottom left corner of the domain. Khiadani^24^ reported that flow became fully established at *x* = 4.575 m from the inlet to the channel. In this research, a 3.312 m long section of the channel was utilized, corresponding to *x* = 4.188–7.5 m in the physical model. All 16 nozzles in the inflow region were retained allowing a distance of 0.5 m and 0.939 m upstream and downstream, respectively. This allowed fully developed flow to be re-established before and after the inflow region, respectively. Thus the inlet at *x* = 0 m was 0.514 m upstream of the centre of the first nozzle while the outlet at *x* = 3.312 m was 0.953 m downstream of the centre of the last nozzle. The primary study was the interaction of the jet with the cross-stream; to reduce the complexity of the model flow within the nozzle was not simulated. Fully developed flow conditions were applied at nozzle exit to match the physical model. Jets were discharging at a constant height of 0.08 m from the bottom of the channel similar to the physical model; the upper region was filled with air and the total height of the domain was 0.09 m. Taking advantage of symmetry in the *x*–*y* plane at the centreline, one half of the channel was modelled as shown in Fig. 1.

### Flow solver

The numerical package FLUENT version 17.2^69^ was used to carry out simulations. The volume of fluid was used to model two-phase flow. A pressure based solver applicable for a wide range of flow speed and both compressible and incompressible flows was used^70–72^. The PISO (Pressure-Implicit with Splitting of Operators) solution algorithm which is often employed in simulations involving large time steps and high degree of mesh distortion^73^ was applied. The spatial discretization scheme used in the current study was the second order upwinding method.

**Figure 2:**
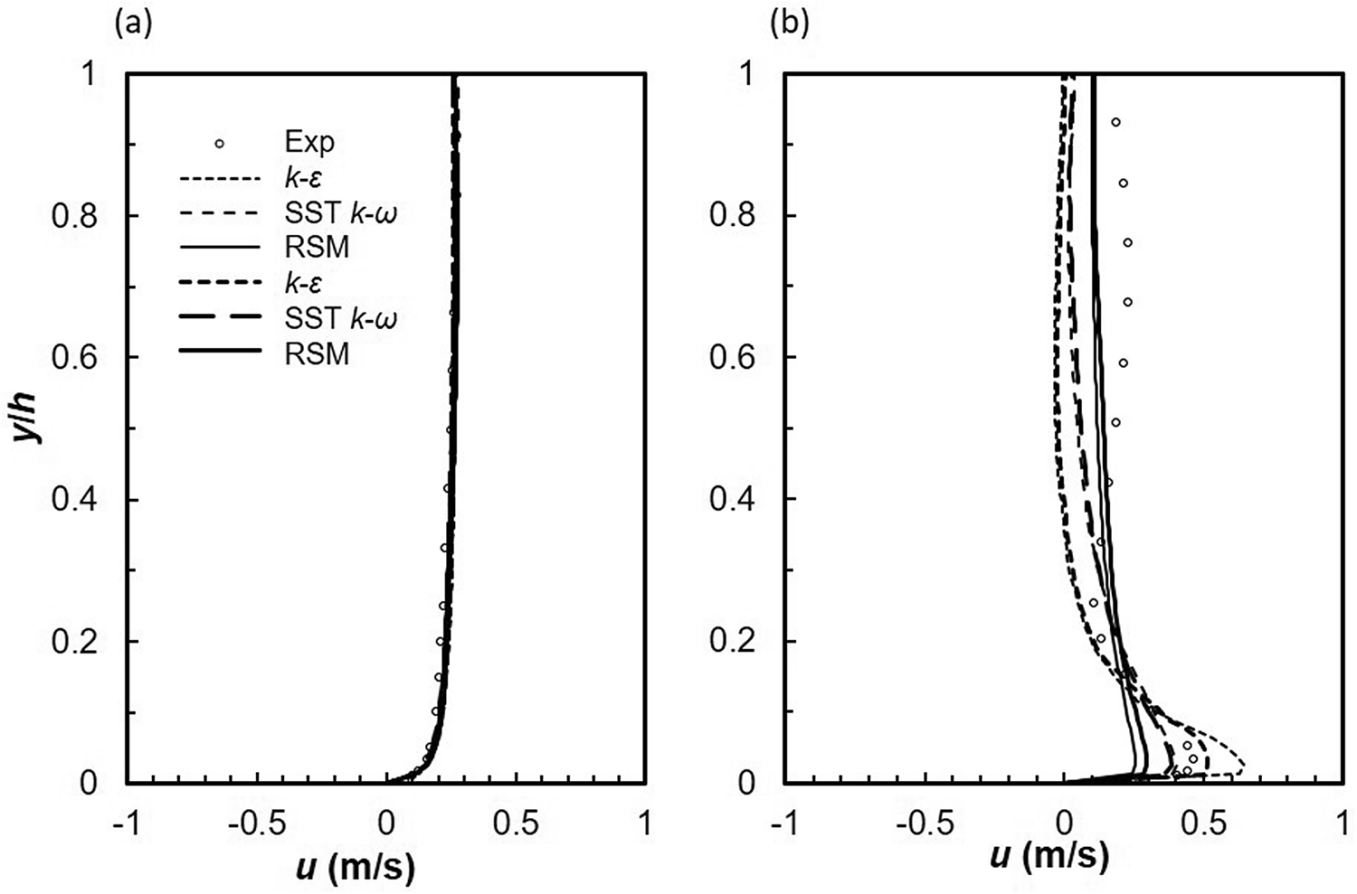
Effect of grid refinement on longitudinal velocity at (**a**) *x* = 4.575 m (**b**) *x* = 5.255 m (Line size 1, Grid 1 and Line size 1.5, Grid 2).

An unstructured mesh composed of hexahedral elements within the core and inflation prism layers on the walls namely the channel bed and side wall was generated by ANSYS Mesher version 17.2^34,68,74^. Refinement was applied to the inflow region where rapid flow variation was expected. Inflation at the wall consisted of twenty layers; the height of the first cell was 0.07 mm corresponding to a *y*^+^ value approximately equal to 1 at the wall. Within the core, the Kolmogorov length scale *η* was used to determine the appropriate cell size. To establish a grid independent solution, five grid sizes were generated ranging from 0.8 × 10^6^ to 4.8 × 10^6^ cells. Figure 2 shows profile plots of longitudinal point velocity for converged solutions of Grid 1 and Grid 2 with 1.61 × 10^6^ and 2.03 × 10^6^ elements, respectively at two locations, one before the lateral inflow region and the second approximately at the centre of the inflow zone. Minimum differences were noted; therefore, Grid 1 mesh was used to minimize computation time. Due to reasonable agreement between each model with experimental data the same mesh was used for all three models. In addition, experimental data was used for boundary and initial conditions to further improve performance *k*–*ε*, SST *k*–*ω* and RSM models were then run for each lateral inflow rate with an appropriate time step size based on the Courant-levy criterion^75^.

### Initial and boundary conditions

Due to the slope, the bottom level varied from 0.009936 m at the inlet to 0.0 m at the outlet. Fully developed flow profiles were specified at the inlets for both cross-flow and the jets based on experimental results. At both outlets i.e. for crossflow and for ambient air above the body of water, pressure-outlet boundaries were specified. Gauge or static pressure must be specified at a pressure outlet boundary. At the channel outlet gauge pressure was set to zero; hydraulic diameter, water depth and turbulence intensity were also specified based on experimental data. Hydraulic diameter was determined from measured water depth and the geometry of the physical model. At the ambient outlet, gauge pressure was set to zero and volume fraction of air to 1 since only one phase, i.e. air is present. At solid boundaries velocities were assumed to be zero and smooth and a no slippery boundary condition selected. The fluxes of all quantities were assumed equal to zero across a symmetry boundary; normal velocity and gradients of all scalar quantities equal to zero.

## Results and discussion

For this work, the solution was considered converged when all residuals were lower than 1 × 10^–4^, and monitors of average velocity upstream, downstream and within the inflow region were constant. In addition, the net flux imbalance was within 10% of the minimum flux into the domain^68,69^. Although results were obtained for two lateral inflow rates, due to page restrictions, only the results for *q* = 2.1.4 L s^−1^ m^−1^ are presented here. Further details can be found in Chipongo^76^.

### Velocity distributions

**Figure 3:**
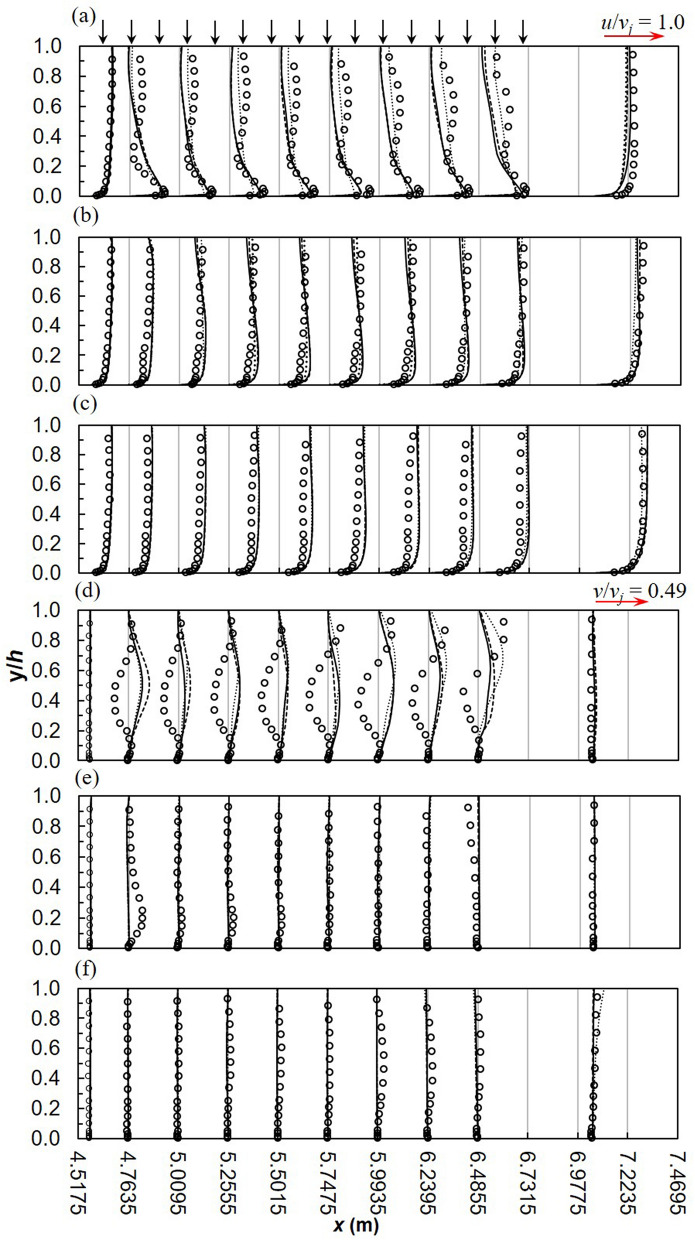
Longitudinal velocity profiles, *u*/*v*_*j*_ at (**a**) *z* = 0 m (**b**) *z* = 0.075 m (**c**) *z* = 0.15 m and vertical velocity profiles, *v*/*v*_*j*_ at (**d**) *z* = 0 m (**e**) *z* = 0.075 m (**f**) *z* = 0.15 m for *q* = 2.14 L s^−1^ m^−1^ (dashed line, realizable *k*–*ε*; solid line, SST *k*–*ω*; dotted line, RSM; markers-experimental data).

It is common practice, in open channel flow to present the velocity and Reynolds stresses in dimensionless form by dividing by the shear velocity, u^*^. In fact, Nezu and Nakagawa^48^ refer to u^*^ as a fundamental velocity scale for normalizing turbulence characteristics. Such a scaling is based on the validity of the log-law in open channel flows and has proven useful in determining empirical relationships for open channel flow. Khiadani et al.^6^ concluded that the log law is invalid in the lateral inflow region, as a result the jet velocity *v*_*j*_ was used to normalize turbulence characteristics. Experimental and numerical stream-wise *u* velocity profiles normalized by the jet velocity, *v*_*j*_ are shown in Fig. 3a-c. In general, good agreement exists between experiments and numerical models. In the stream-wise direction, numerical models are an excellent match to experimental data in the uniform flow region upstream of the lateral inflow region and at *z* = 0.075 m in the span-wise direction. Downstream of the lateral inflow region, numerical models underestimate velocity profiles, although the general shape is in agreement with experiments. Worth and Yang^30^ partly attributed this discrepancy to the coarser grid downstream of the jet impingement point, which applies to this research. The same grid size was used for both uniform flow regions upstream and downstream of the lateral region; refinement was only applied to the lateral inflow region. In the vertical direction, numerical models are a closer match to experiments within the wall jet (0 < *y*/*h* ≤ 0.03) and the region above it characterized by negative velocity gradients (0.03 < *y*/*h* ≤ 0.2), apart from the RSM which under-predicts the magnitude of the wall jet. The inability of RANS to model the wake region behind the jets is well established^77^. On the other hand, consistently with the findings of Worth and Yang^30^, Ostheimer and Yang^29^ and Galeazzo et al.^77^, Fig. 3a-c shows good performance of both realizable *k*–*ε*, and SST *k*–*ω* models in the wall region. Accurate prediction of the magnitude of velocity of the wall jet is essential for the precise prediction of the length of the ground vortex^30^. Moreover this characteristics affects skin friction^30,31^. However, at the channel centreline, numerical models fail to predict the general shape of the experimental profile especially within the bulk of fluid flow (see Fig. 3a). In this region (*y*/*h* > 0.2), numerical models significantly deviate from experiments; however, the RSM is slightly more comparable. Chakraborty^78^ reported better performance from both realizable *k*–*ε*, and SST *k*–*ω* models over the RSM at a velocity ratio of 2 for a similar arrangement but with a single jet. Due to inflow addition, crossflow velocity varies across the channel leading to a decrease in velocity ratio. Table 2 shows that velocity ratio is less than 2. Thus the conclusion of Chakraborty^78^ is valid, although the interaction of the wall jets on consecutive jets is significant.

Vertical velocity profiles are illustrated in Fig. 3d-f. Numerical profiles are almost a mirror-image of experimental at the channel centreline with the former predicting positive and the latter negative values of *v* velocity. In this region, experimental and numerical velocity vectors are downward and upward, respectively. Moreover, the magnitude of vertical velocity predicted by numerical models is much lower. Such large deviations were also noted by Worth and Yang^30^ and Ostheimer and Yang^29^ for a single and twin impinging jets, respectively. However, Ostheimer and Yang^29^ numerically predicted negative values similar to experiments. It must be noted that the velocity ratio in the study of Ostheimer and Yang^29^ was 30, with a crossflow velocity of 0.18 m s^−1^. The jet was not deflected or “bent-over” by the crossflow, as a result a fountain or up-wash vortex was formed between the jets. In this research rapid deflection of the jet by the crossflow weakens its strength at impact with the channel bed, no fountain vortex was observed. Worth and Yang^30^ conducted their research in similar conditions but with a single jet. In the uniform flow region upstream and downstream of the inflow zone, numerical models match experiments. Chakraborty^78^ noted a similar trend for *r* = 2 further downstream of the impingement point however, the bulk of data matched experiments. In this study, due to the proximity of the jets, the ground vortex due to each jet is possibly attached to the leading jet causing significant flow obstruction. As a result, there is less fluid in the wake region, thus fluid from the wall jet is sucked or lifted up resulting in the positive vertical velocity. Clearly such a phenomenon was not observed in the experiments of Khiadani^24^. A possible explanation is that due to the close proximity of the jets, RANS models fail to accurately predict the trailing edge location of ground vortex thus predicting a semi-detached instead of a fully detached vortex. RANS models are unable to simulate wake structures or separation points in flow around circular cylinders. In addition, narrower wakes are predicted thus resulting in a thicker ground vortex^79^.

**Figure 4:**
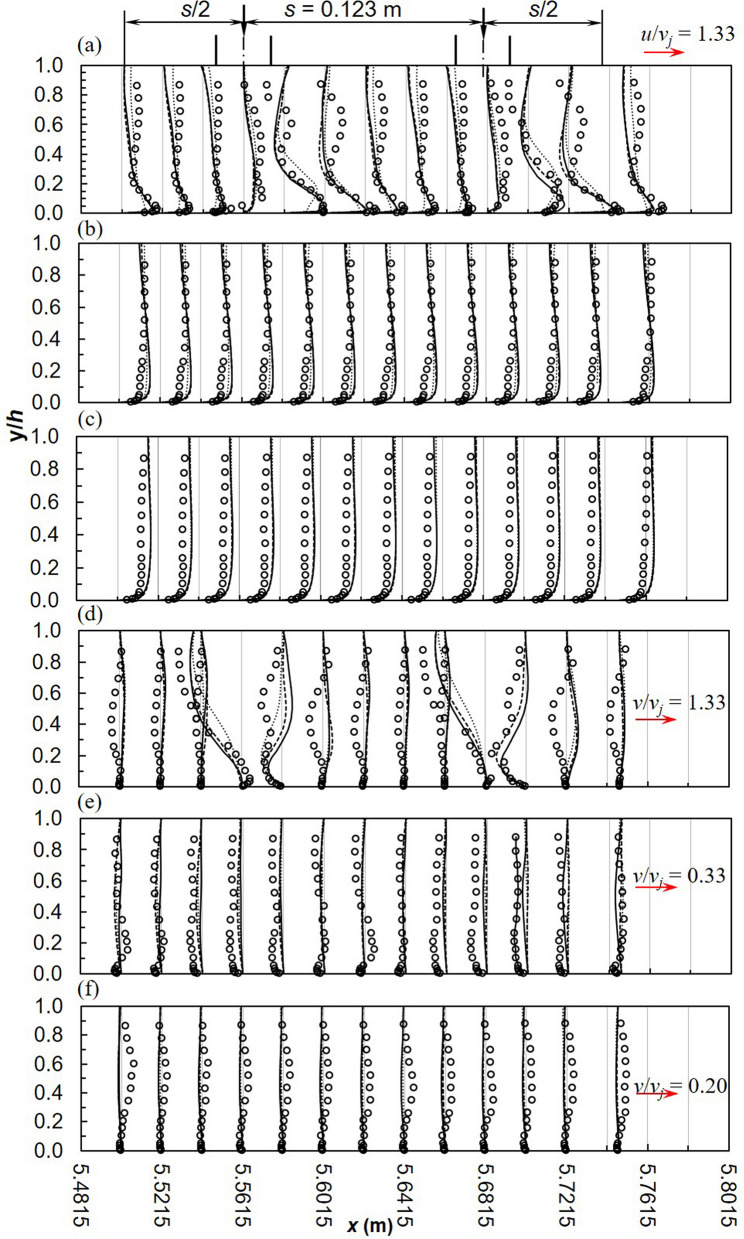
Between 2 jets, stream wise velocity profiles, *u*/*v*_*j*_ (**a**) *z* = 0 m (**b**) *z* = 0.075 m (**c**) *z* = 0.15 m and vertical velocity profiles, *v*/*v*_*j*_ (**d**) *z* = 0 m (**e**) *z* = 0.075 m (**f**) *z* = 0.15 m (dashed line, realizable *k*–*ε*; solid line, SST *k*–*ω*; dotted line, RSM; markers-experimental data).

Khiadani^24^ described flow characteristics between two consecutive nozzles highlighting the formation of the wall jet parallel to the bed, downstream of the jet. The impinging jet induces negative *u* values upstream of the nozzles from the bed to approximately *y*/*h* < 0.05. Near the bed, the momentum of the advancing crossflow deflects the jet. The impinging jet induces fluid draw down near the free surface downstream causing the fluid around to be sucked in creating a recirculating zone whose strength increases downstream. The comparison of numerical and experimental stream-wise *u*/*v*_*j*_ velocity between consecutive jets is displayed in Fig. 4a-c. A good match is observed between numerical models and experiments, particularly at *z* = 0.075 m. The realizable *k*–*ε* and SST *k*–*ω* models predict similar values which are closer to experiments. RSM underestimates the magnitude of the wall jet near the bed of the channel as well as velocity profiles in close proximity of the impinging jet at the symmetry plane. This was also observed by Worth and Yang^30^ and Chakraborty^78^. Near the water surface, the RSM performs much better (*y*/*h* > 0.5). According to Gibson and Rodi^80^, turbulence is highly anisotropic near the water surface therefore RSM with modifications for free surface effects are necessary. All RANS models fail to accurately predict the length of the reverse flow region on the lee-side of jet impingement point due to the limitations mentioned earlier. This flaw is critical in the development of turbulence structures further downstream from the jet.

For the same location, vertical velocity *v*/*v*_*j*_ between consecutive jets is shown in Fig. 4d-f. At impingement, numerical models correctly predict the magnitude and direction of the jet. In this region, flow is downward due to the impinging lateral inflow. RSM slightly miscalculates the magnitude of lateral inflow. Further downstream, the uplifting of the wall jet characterised by positive vertical velocity is apparent on the lee-side of the jet. This characteristic is consistent along the cross-section at *z* = 0.075 m and 0.15 m although due to the small magnitudes of *v* in these locations, this is not apparent from Fig. 4e-f.

**Figure 5:**
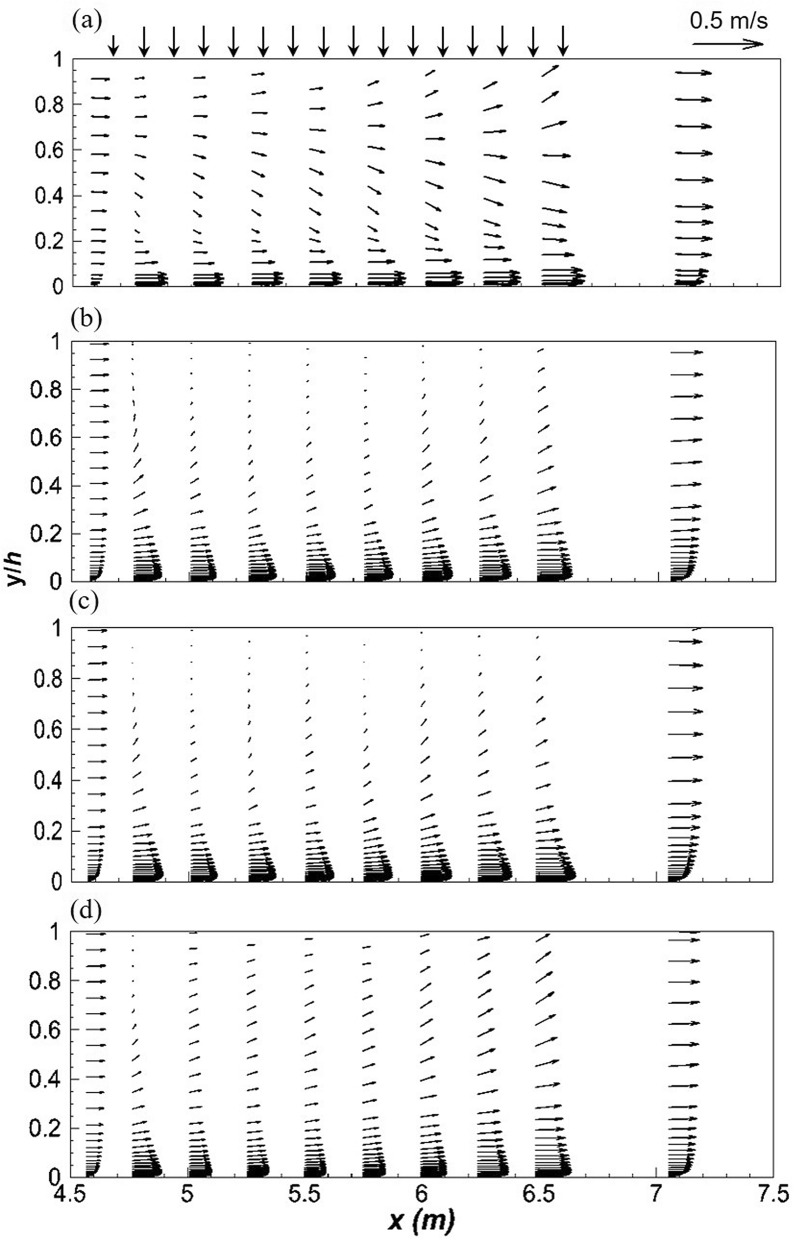
*u*–*v* vector plots along the centre line of the channel (**a**) experiments (**b**) realizable *k*–*ε* (**c**) SST *k*–*ω* (**d**) RSM.

Figure 5 shows *u*-*v* velocity vectors along the symmetry plane. Results from numerical models match experiments before and after the lateral inflow region. This implies that RANS models are suitable for modelling uniform open channel flow. Visible differences between the numerical models and experiments are the upward vectors from approximately *y*/*h* = 0.1 at the start of the lateral inflow region. Subsequently, the wall jet is lifted resulting in a thicker wall jet compared to experiments. As a result, more blockage occurs upstream of the adjacent jet and fluid flow is upward. In this study the effect of the free surface is significant; RSM performs best in this region. The shortcoming of the *k*–*ε* and SST *k*–*ω*, is their inability to account for history of flow upstream which influences motions of the larger scale eddies and contributes to the transfer of scalar quantities such as turbulent energy^48^.

**Figure 6:**
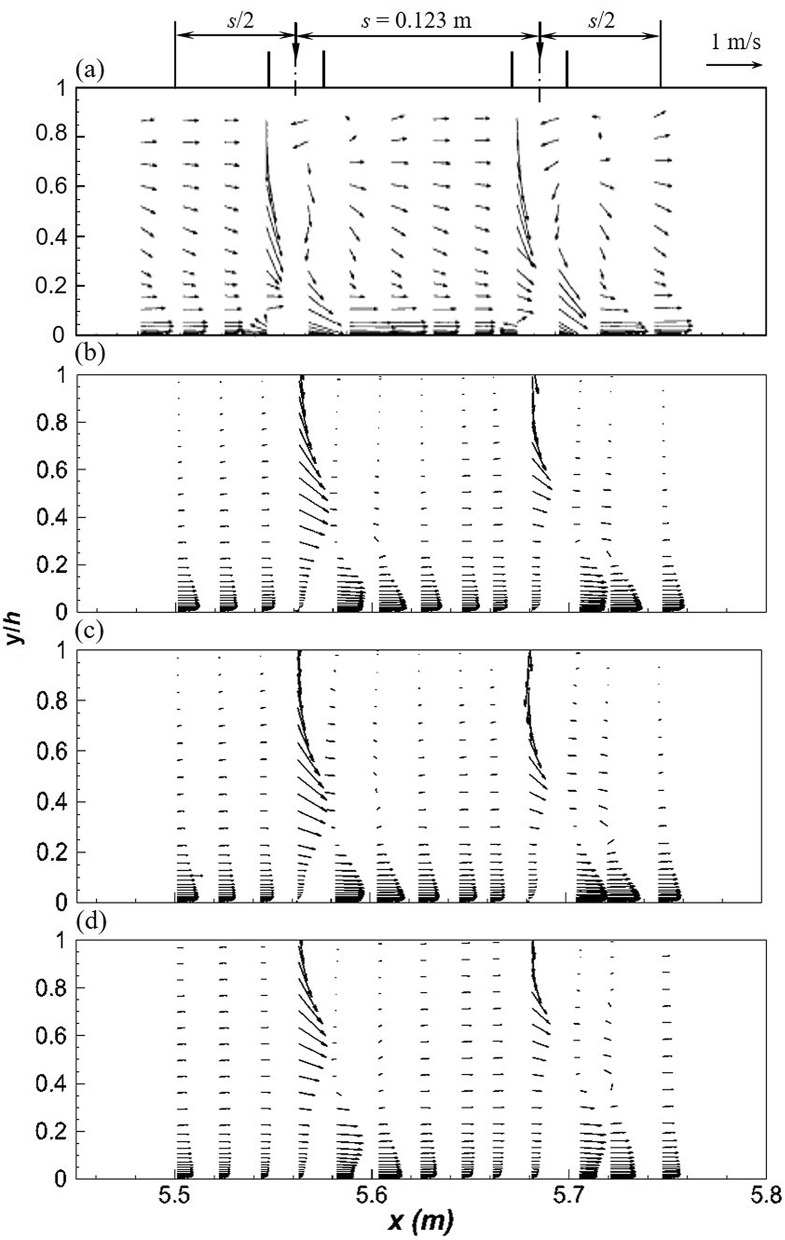
*u*–*v* vector plots along the centerline of the channel between 2 jets for *q* = 2.14 L s^−1^ m^−1^ (**a**) experiments (**b**) realizable *k*–*ε* (**c**) SST *k*–*ω* (**d**) RSM.

Between two jets, *u*–*v* vector plots along the centreline of the channel between 2 jets are shown in Fig. 6, respectively. The realizable *k*–*ε* and SST *k*–*ω* predicted reverse flow at the impingement point but underestimate both the height and magnitude; RSM failed to capture any reverse flow. According to experiments, the reverse flow penetrates to about *y*/*h* = 0.05 from the bed of the channel and about *y*/*h* = 0.025 for the realizable *k*–*ε* and SST *k*–*ω*. The maximum reverse flow from experiments is approximately 0.12 m s^−1^ and 0.018 m s^−1^ for the realizable *k*–*ε* and SST *k*–*ω*, respectively. Midway between the jets, RSM predicts velocity vectors analogous to the uniform flow region, implying that flow is almost parallel to the channel bed, with minimal influence from the impinging jet. This is a critical flaw since experiments clearly show that flow between consecutive jets is significantly affected by the impinging jet.

Vector plots clearly show the formation of vortices in front of the jet, from about *y*/*h* = 0.2 to 0.6. The predicted location of the centre of the vortex varies amongst turbulence models. Measurements were taken every 20 mm therefore the centre of the vortices cannot be accurately calculated. However, it can be implied from Fig. 6 that the centre of the vortex in the subsequent jet is lower than the leading due to decrease in flow depth along the channel. In studies of multiple tandem jets in crossflow, the centre of the vortex in the second jet was lower due to the shielding effect of the leading jet allowing it to penetrate deeper into the crossflow; from the second jet, the centre of the vortex remained consistent^81,82^.

### Turbulence characteristics

In this section turbulence intensity and Reynolds stresses are discussed. Experimentally, Reynolds stresses are determined from measurements of instantaneous velocity in all three dimensions—their estimation is detailed in Khiadani^24^. For RANS models from the two-equation family, Reynolds stresses are computed from the eddy viscosity, *μ*_*t*_, determined from the Boussinesq approximation^44^. RSM directly approximates Reynolds stresses from flow properties hence the six Reynolds stresses can be extracted from FLUENT (v. 17.2)^69^. For comparison with LDA measurements, the three components of turbulence intensity and the Reynolds shear stress are estimated from the following equations^83^:

3 $$ U^{\prime} = \sqrt {\overline{{u^{\prime}u^{\prime}}} } = \sqrt {\frac{{\mu_{t} \left[ {\frac{\partial u}{{\partial x}} + \frac{\partial u}{{\partial x}}} \right] - \frac{2}{3}\left[ {\rho k + \mu_{t} \frac{\partial u}{{\partial x}}} \right]}}{ - \rho }} $$

4 $$ V^{\prime} = \sqrt {\overline{{v^{\prime}v^{\prime}}} } = \sqrt {\frac{{\mu_{t} \left[ {\frac{\partial v}{{\partial x}} + \frac{\partial v}{{\partial x}}} \right] - \frac{2}{3}\left[ {\rho k + \mu_{t} \frac{\partial v}{{\partial x}}} \right]}}{ - \rho }} $$

5 $$ U^{\prime}V^{\prime} = \overline{{u^{\prime}v^{\prime}}} = \frac{{\mu_{t} \left[ {\frac{\partial u}{{\partial y}} + \frac{\partial v}{{\partial x}}} \right]}}{ - \rho } $$

Velocity gradients *∂u*/*∂x*, *∂v*/*∂x*, *∂u*/*∂y*, and *∂v*/*∂x*, turbulence kinetic energy *k*, and eddy viscosity*, μ*_*t*_ are direct FLUENT (v. 17.2)^69^ outputs while *ρ* is the density of water.

**Figure 7:**
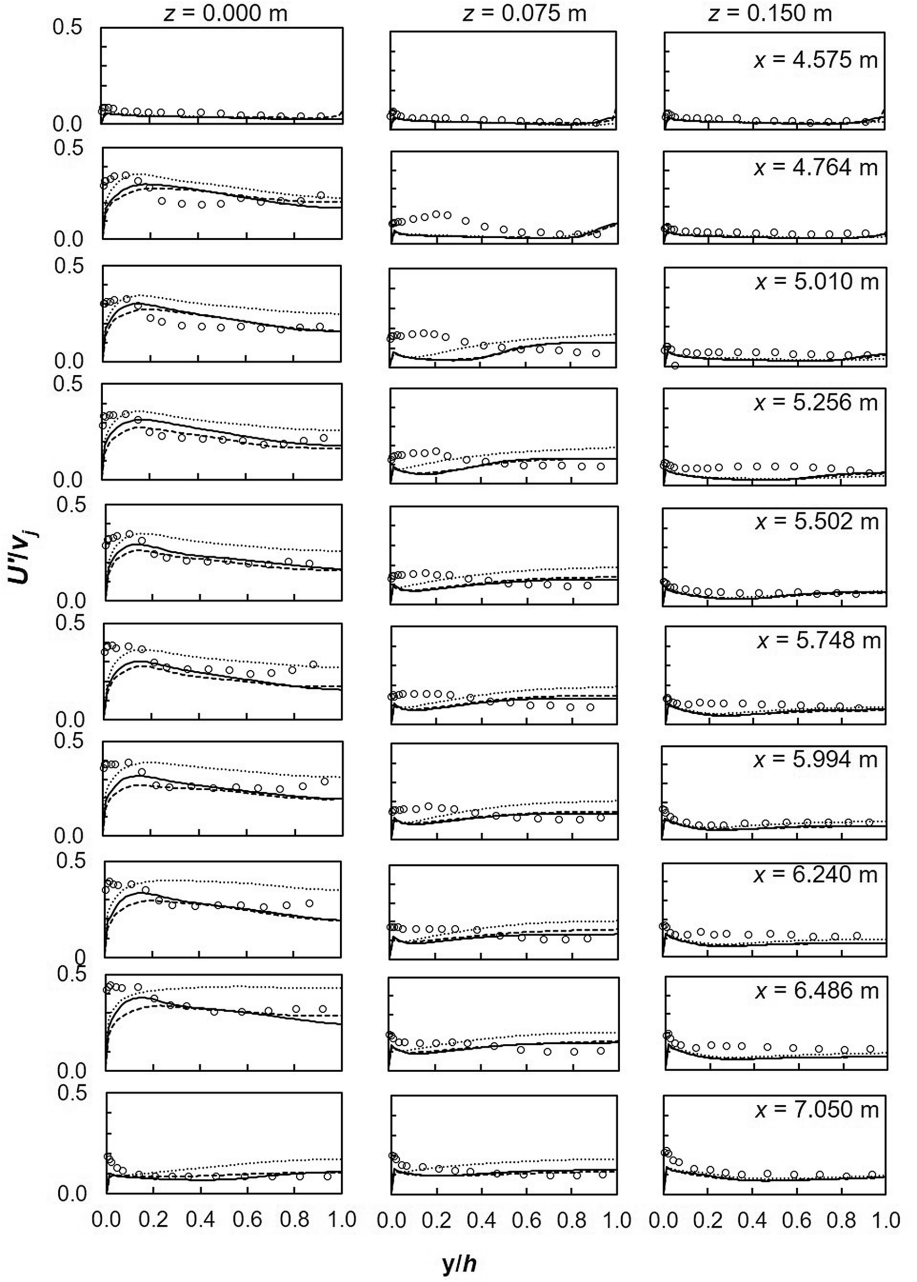
Turbulence intensity *U′*/*v*_*j*_ (dashed line, realizable *k*–*ε*; solid line, SST *k*–*ω*; dotted line, RSM; markers-experimental data).

A comparison of stream-wise turbulence intensity *U′*/*v*_*j*_ from both experiments and turbulence models is illustrated in Fig. 7. Along the cross-section, models are a closer match to experiments away from the symmetry plane; the best results were observed closest to the wall where the effect of impinging jets is reduced. RANS models underestimate *U*′/*v*_*j*_ near the bed across the cross-section. Moreover, the SST *k*–*ω* and the realizable *k*–*ε* are a good match with experiments in the uniform flow region upstream and downstream of the lateral inflow region. In this region, no recirculation zones, wakes or vertices exist and streamlines are parallel to the channel bed. Minor differences exist between the SST *k*–*ω* and the realizable *k*–*ε* although the latter is slightly closer to experiments at approximately *y*/*h* < 0.5. RSM performs the least along the symmetry plane and worsens downstream; *U′*/*v*_*j*_ near the free surface is overestimated, besides being consistently closer to experiments near the bed (at *y*/*h* < 0.2). Close to the bed, where maximum *U′*/*v*_*j*_ values are recorded, the RSM only predicted half the experimental value^30^. In another study, large deviations were noted between the RSM and experiments, moreover general profiles did not match^29^. Such over estimations were attributed to poor transport and diffusion modelling and the breakdown of assumptions in the high curvature impingement region. Likewise, Chakraborty^78^ observed better performance with the realizable *k*–*ε* and SST *k*–*ω* models, the RSM failed to accurately predict *U′*/*v*_*j*_.

**Figure 8:**
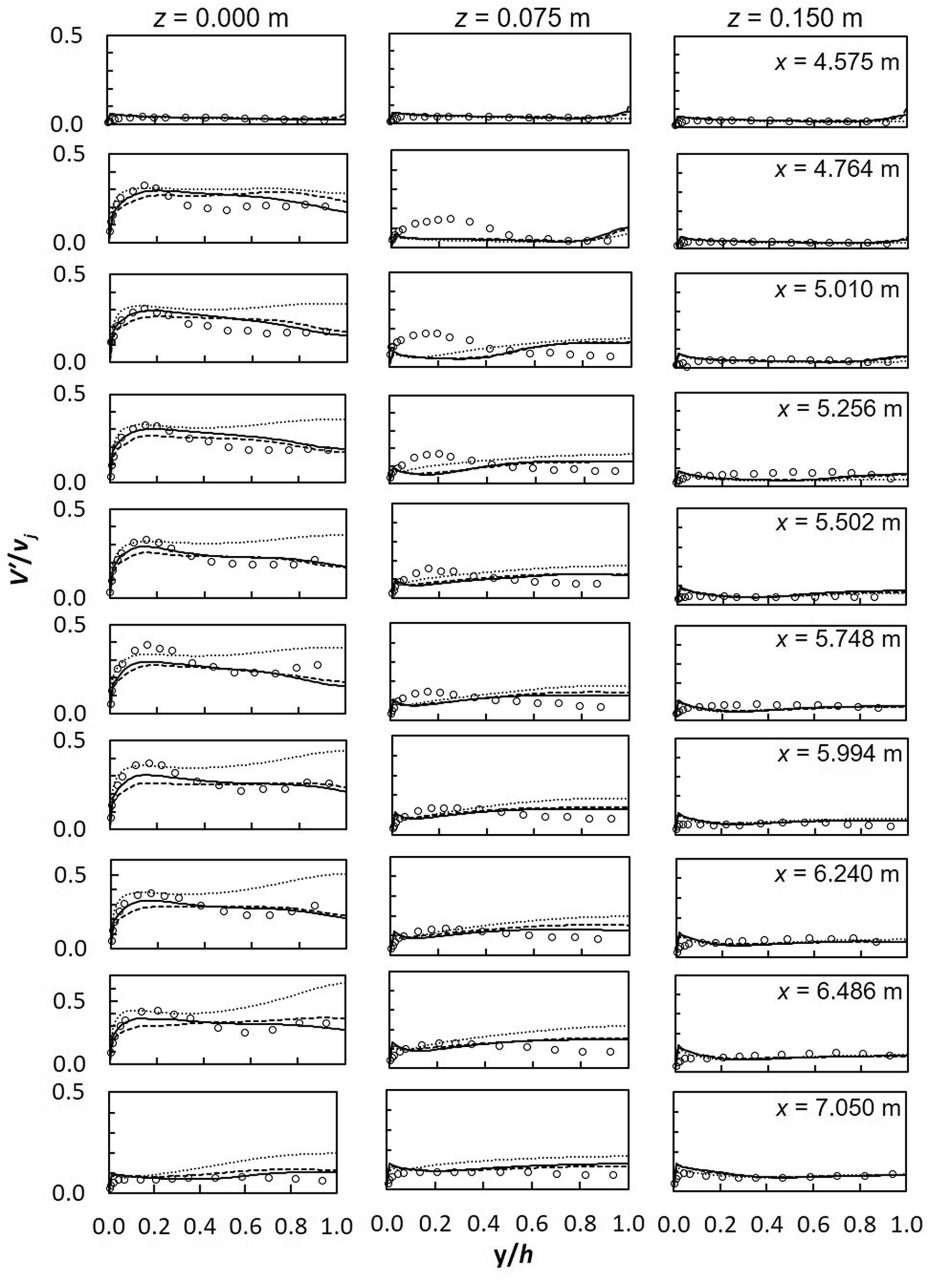
Turbulence intensity *V′*/*v*_*j*_ (dashed line, realizable *k*–*ε*; solid line, SST *k*–*ω*; dotted line, RSM; markers-experimental data).

Vertical turbulence intensity is illustrated in Fig. 8. SST *k*–*ω* models is more comparable to experiments although profiles have different shapes. Towards the side-wall, minor differences exist among turbulence models; with the best match between models and experiments observed closest to the side-wall (*z* = 0.15 m) possibly due to the reduced effect of impinging jets. RANS models over-predict *V′*/*v*_*j*_ near the bed and near the free-surface due to the wall jet uplifting observed downstream of jet impingement and free-surface effects, respectively. At the symmetry plane, results from SST *k*–*ω* and realizable *k*–*ε* are similar; RSM increasingly overestimates experiments. In a study of a single jet in confined crossflow, large deviations were observed between RSM and experiments particularly near the confinement wall which decreased downstream ^84^. The progressive movement of the semi-detached wall jet continues to block more flow on the upstream side of the jet thus causing the development of complex flow structures downstream.

**Figure 9:**
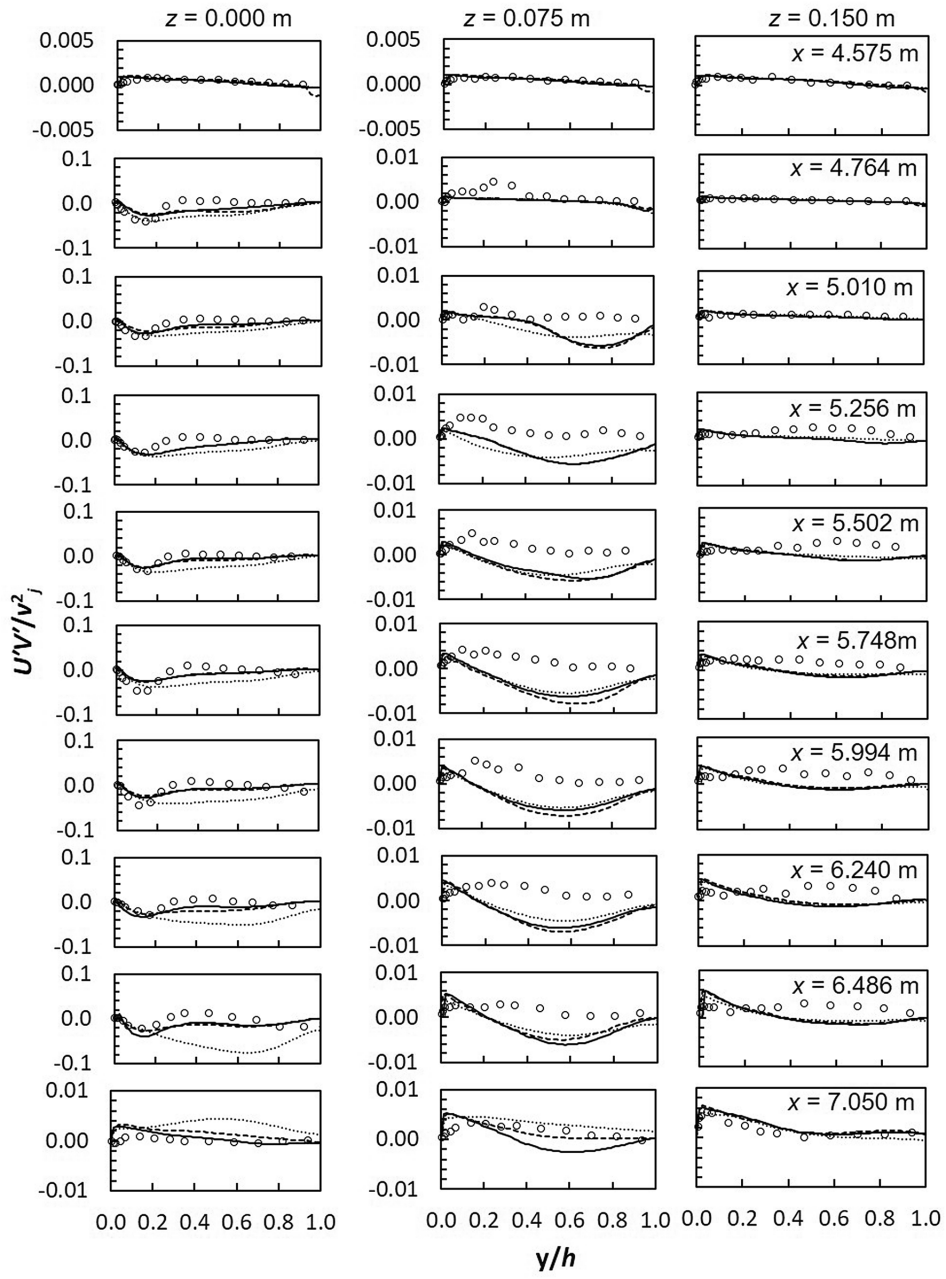
Reynold shear stresses *U′V′*/*v*^2^_*j*_ (dashed line, realizable *k*–*ε*; solid line, SST *k*–*ω*; dotted line, RSM; markers-experimental data).

Predicted Reynolds stresses, *U′V′*/*v*^2^_*j*_ are shown in Fig. 9. Numerical models predict slightly more appropriate values in the uniform region upstream and downstream of the lateral inflow region and towards the side-wall where effects of impinging jets are reduced. This is expected because RANS models satisfactory predict turbulence features in simple streamlined flows such as the uniform flow region. However downstream of the lateral inflow region the RSM consistently overestimates Reynolds stresses. In addition, profiles of predicted Reynolds stresses are analogous to experiments. RSM underestimates Reynolds shear stresses at the symmetry plane while SST *k*–*ω* and realizable *k*–*ε* perform much better. At *z* = 0.075 m, numerical models predict the opposite sign for the Reynolds stresses from about *y*/*h* = 0.1. This is the height around which numerical models predict negative vertical velocity instead of the positive depicted by measurements.

**Figure 10:**
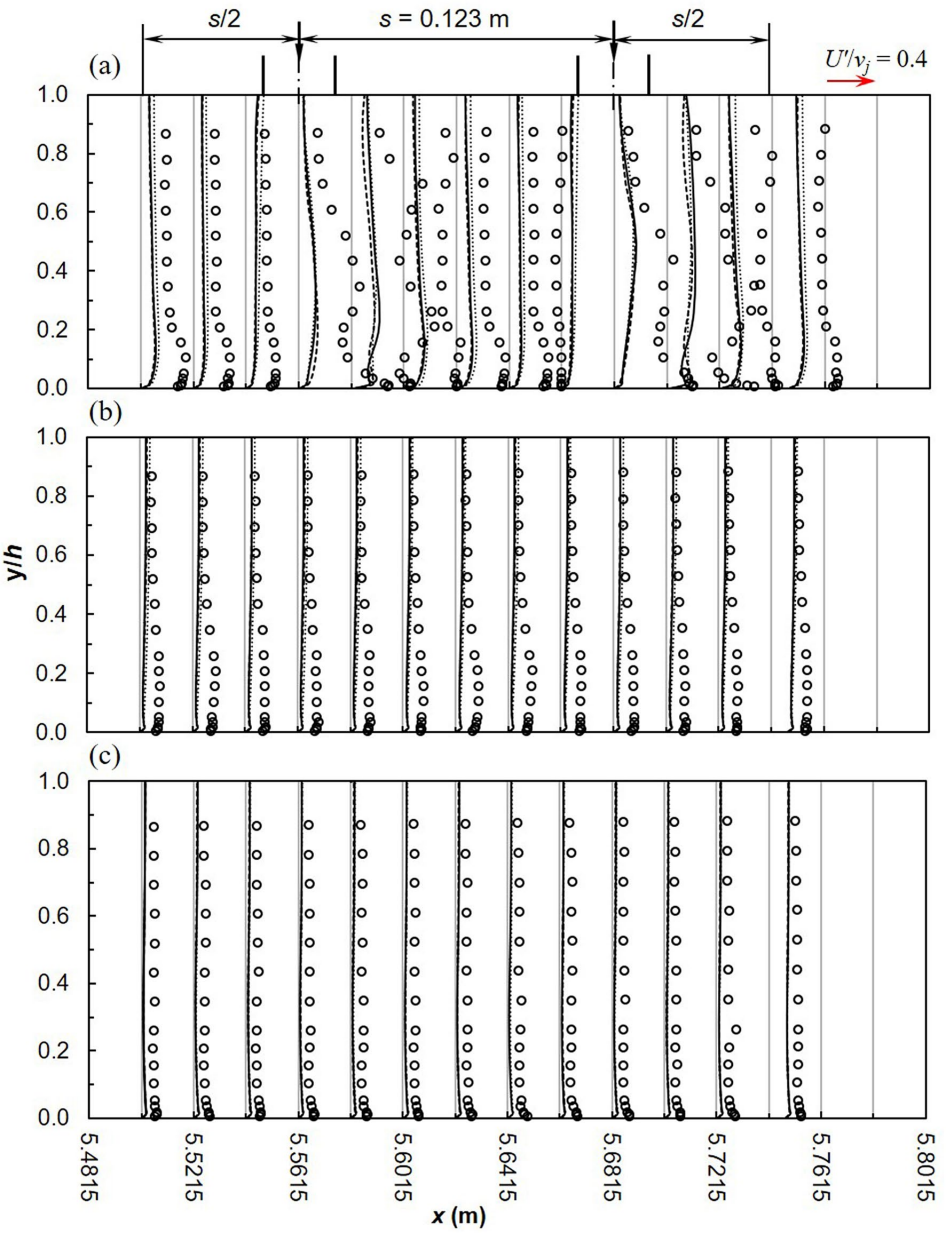
Turbulence intensities, *U′*/*v*_*j*_ between 2 jets (**a**) *z* = 0 m (**b**) *z* = 0.075 m (**c**) *z* = 0.15 m (dashed line, realizable *k*–*ε*; solid line, SST *k*–*ω*; dotted line, RSM; markers-experimental data).

**Figure 11:**
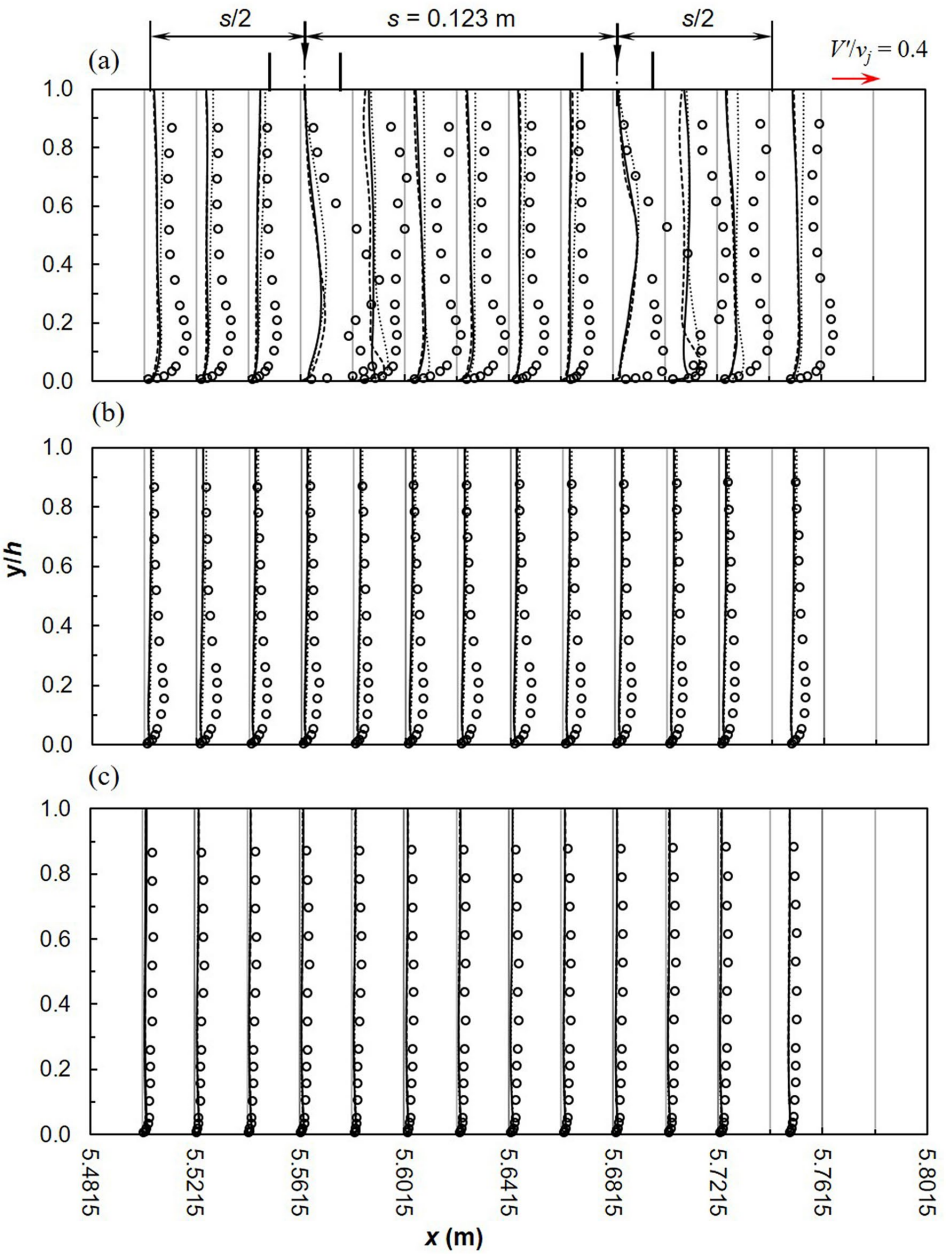
Turbulence intensities, *V′*/*v*_*j*_ between 2 jets (**a**) *z* = 0 m (**b**) *z* = 0.075 m (**c**) *z* = 0.15 m (dashed line, realizable *k*–*ε*; solid line, SST *k*–*ω*; dotted line, RSM; markers-experimental data).

Turbulence characteristics *U′*/*v*_*j*_ between two jets are illustrated in Fig. 10; in general RANS models underestimate experiments. Minor variations exist among the models at the symmetry plane, becoming insignificant towards the wall. Further from the impingement point in the longitudinal direction, deviations between numerical models and experiments diminishes. Demuren^84^ made a similar conclusion based on measurements at 8 and 12 times the jet diameter. Ostheimer and Yang^29^ attributed the poor predictions to flow complexity as well as the formation of unsteady flow features such as ground and fountain vortices. In this study, only ground vortices were formed which had a profound effect on flow development downstream of the jet impingement point. Along the flow depth, largest deviations were observed near the bed and might be due to wall jet uplifting. Vertical turbulence intensities *V′*/*v*_*j*_ are shown in Fig. 11. In general numerical models largely digress from experiments^29,31^. RSM is marginally a better match to experiments particularly near the free-surface along the symmetry plane. Downstream of the first jet, the difference between numerical and experimental profiles decreases^84^. In terms of magnitude there are minor differences among the models, even though according to Worth and Yang^30^ can have significant effects on the flow field.

**Figure 12:**
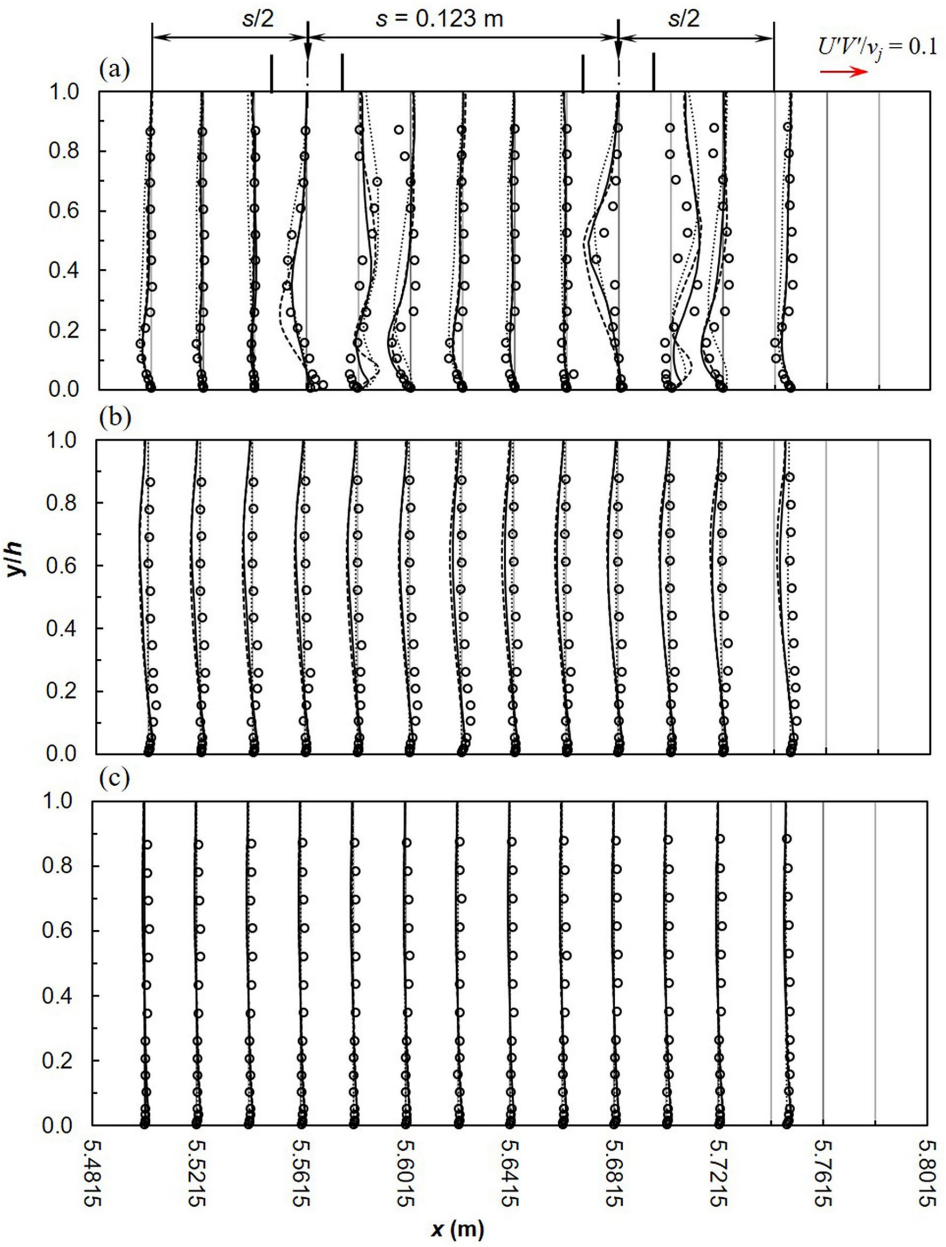
Reynold shear stress, *U′V′*/*v*^2^_*j*_ between 2 jets (**a**) *z* = 0 m (**b**) *z* = 0.075 m (**c**) *z* = 0.15 m (dashed line, realizable *k*–*ε*; solid line, SST *k*–*ω*; dotted line, RSM; markers-experimental data).

Reynold shear stress between two jets are given in Fig. 12. In comparison with *U′*/*v*_*j*_ and *V′*/*v*_*j*_, *U′V′*/*v*^2^_*j*_ is better estimated by numerical models. Good agreement is apparent between the realizable *k*–*ε* and SST *k*–*ω* models in the uniform flow region. RSM underestimates *U′V′*/*v*^2^_*j*_ in this region but remarkably predicts values much closer to experiments at *z* = 0.075 m. The realizable *k*–*ε* and SST *k*–*ω* models underestimate experiments within 0.2 < *y*/*h* < 0.9 at *z* = 0.075 m^30^. Barata et al.^31^ concluded that the poor prediction of Reynolds shear stresses by the standard *k*–*ε* model was not influenced by numerical computations but was rather an indication that the turbulence viscosity hypothesis is not appropriate in the impingement zone.

## Summary and conclusions

**Figure 13:**
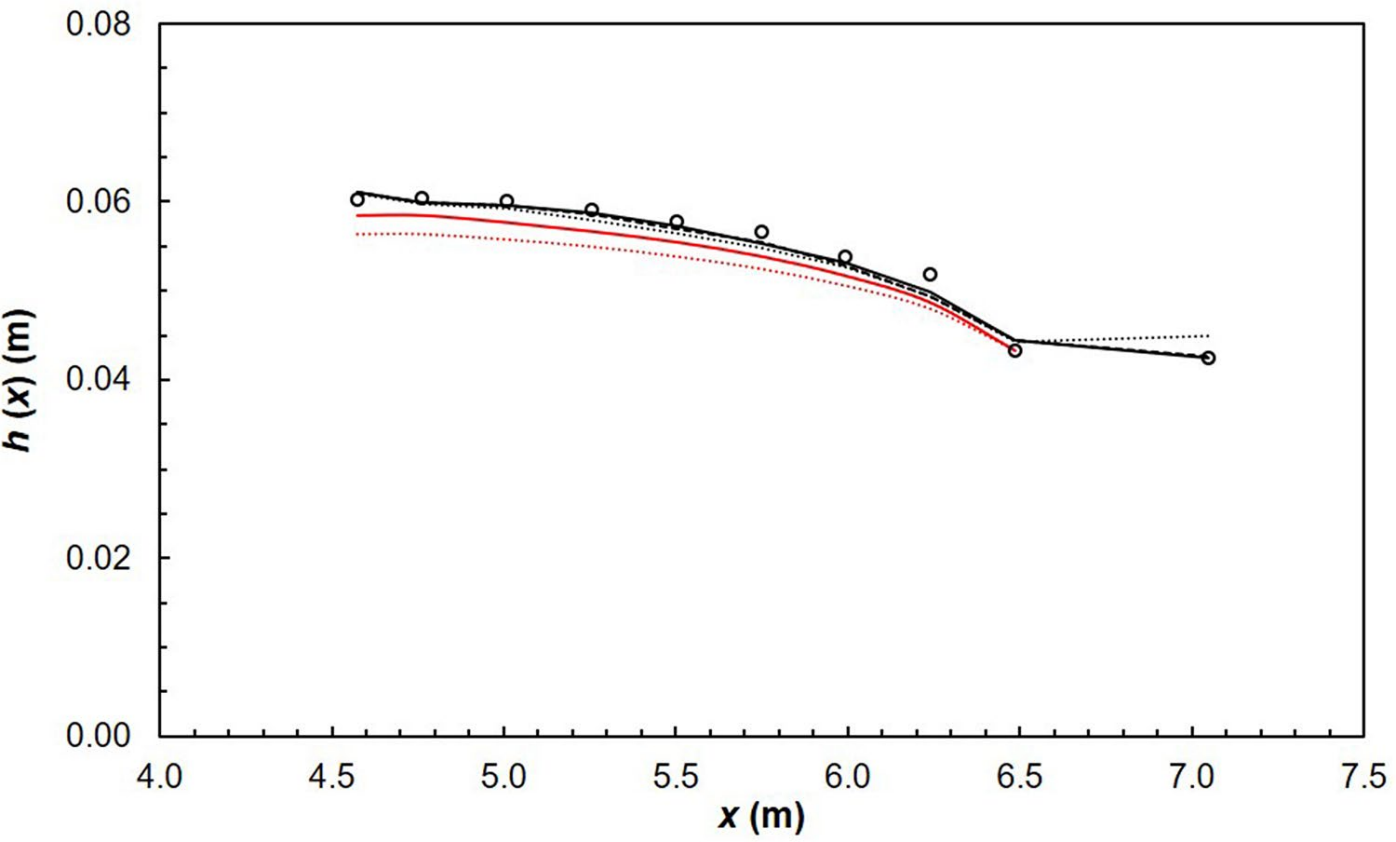
Average water depth in the channel at *z* = 0.075 m (dashed line, realizable *k*–*ε*; solid line, SST *k*–*ω*; dotted line, RSM; markers-experimental data; solid red line, modified SVF equation; dotted red line, original SVF equation).

Predicting the flow depth that occurs in a channel is of particular importance to engineers^10,18,19,85,86^. Limitations of the current one-dimensional approach were discussed in the introduction. In order to investigate whether numerical models are more appropriate, predicted water surface profiles (WSPs) were compared with experiments. For numerical models, the water surface was considered to be the surface in which the void fraction of water *α*_*w*_ = 0.5^87^. Figure 13 shows good agreement between experimental and numerical data. RSM slightly overestimates SST *k*–*ω* and realizable *k*–*ε* downstream of the lateral inflow zone thus predicting more flow bulking. In addition, WSPs were also computed using both the original^16–21^ and modified one-dimensional SVF equation^15^. Chipongo and Khiadani^15^ modified the SVF equation to account for flow blockage due to the impinging jets. Figure 13 indicates that the original equation underestimates measured WSPs while the modified SVF equation is a closer match to experiments and the more complex numerical models. Therefore, to estimate WSPs for design purposes, considering the insignificant differences between complex numerical models and the modified SVF equation, it is more efficient to use the latter.

The aim of this paper was to establish if turbulence models were capable of matching SVF experimental data and to identify the RANS model that gives optimum results. It was observed that all models accurately predicted flow depth often used in the theoretical design of SVF. In terms of predicting velocity distributions and turbulence parameters no turbulence model showed superior overall performance; strengths and weaknesses were noted in each. Realizable *k*–*ε* and SST *k*–*ω* underestimated velocity and turbulence intensities at the symmetry plane but were able to predict the reverse flow that occurs near the bed at impingement. RSM showed no significant improvements from realizable *k*–*ε* and SST *k*–*ω* and failed to accurately predict turbulence intensities despite the additional equations solved.

Many researchers of jets in crossflow have made the same conclusion for the pressure-strain RSM^29,30,78,82^. Considering the heavy computational effort required, RSM showed no advantage over realizable *k*–*ε* and SST *k*–*ω*. This was a preliminary study based on modelling assumptions (e.g. closures), LES and DNS are desired for more detailed turbulent flow prediction. Further studies are necessary to investigate the effects of the negative vertical velocity predicted by RANS, with attention on the effect of spacing between the jets, co-flow velocity as well as the impact of lateral inflow.

## Abbreviations

*A*: Cross-sectional area specified at a point* x* (m^2^)
*A*_0_, *A*_*s*_: Constants of the *k*–*ε* model (−)
*C*: Constant of proportionality in dimensionless form (−)
*C*_*ε,1,*_* C*_*ε,2,*_* σ*_*ε,1*_: Constant associated with the Reynolds stress model (−)
*C*_*μ*_, *C*_*ε1*_, *C*_*ε2*_, *σ*_*k*_, *σ*_*ε*_: Constants for the *k*–*ε* model (−)
*D*_*ω*_: Cross diffusion term (kg m^−^^3^ s^−^^2^)
F: Froude number (−)
*F*: Weighting function in SST *k*–*ω* (−)
$\vec{F}$: Term accounting for external forces (kg m s^−^^2^)
*g*: Acceleration due to gravity (m s^−^^2^)
*G*_*B*_: Buoyancy production term in the *k*–*ε* model (kg m^−^^1^ s^−^^3^)
*G*_*k*_: Generation of turbulent kinetic energy due to mean velocity gradient (kg m^−^^1^ s^−^^3^)
*G*_*ij*_: Buoyancy production term in the Reynolds Stress Model (kg m^−^^1^ s^−^^3^)
*G*_*ω*_: Generation of specific dissipation rate *ω* (kg m^−^^3^ s^−^^2^)
*h*: Water depth at a specific point (m)
*i*, *j*, *m*: Cartesian coefficients (−)
*k*: Kinetic energy (m^2^ s^−^^2^)
*l*: Length scale in turbulence models (m)
$\dot{m}_{pq}$: Mass transfer from fluid* p* to fluid *q* (kg s^−^^1^)
$\dot{m}_{qp}$: Mass transfer from fluid *q* to fluid *p* (kg s^−^^1^)
*N*: Nozzle number (−)
*p*: Pressure
*P*_*ij*_: Generation of the rate of turbulence stress by mean strain/production tensor term (kg m^−^^1^ s^−^^3^)
*q*: Lateral flow rate per unit length of the channel (m^2^ s^−^^1^)
*Q*: Total discharge (m^3^ s^−^^1^)
*r*: Velocity ratio = *v*_*j*_/*U* (−)
Re: Reynolds number (−)
*R*_*h*_: Hydraulic radius given by *A*/*P* (m)
*S*_0_: Slope of the channel with respect to the horizontal plane (−)
$s_{ij} (t)$: Rate of deformation (s^−^^1^)
$s_{ij}^{\prime }$: Fluctuating component of the rate of deformation (s^−^^1^)
*S*_*ij*_: Mean rate of deformation (s^−^^1^)
*S*_*αj*_: Mass source term (kg s^−^^1^)
*t*: Time (s)
*T*: Temperature (K),
*u*, *v*: Mean velocity in *x*-direction, and *y*-direction, respectively (m s^−^^1^)
*u′*, *v′*: Fluctuating components of velocity in the *x*-direction, and *y*-direction, respectively (m s^−^^1^)
*u**: Shear velocity (m s^−^^1^)
$U^{\prime } = \sqrt {\overline{{u^{\prime 2} }} }$: Turbulence intensity in the longitudinal direction (m s^−^^1^)
$$U^{\prime } V^{\prime } = \overline{{u^{\prime } v^{\prime } }}$$: Reynolds stresses (m s^−^^1^)
$V^{\prime } = \sqrt {\overline{{v^{\prime 2} }} }$: Turbulence intensity in the vertical direction (m s^−^^1^)
*v*: Kinematic viscosity (m^2^ s^−^^1^)
*v*_*j*_: Jet exit velocity (m s^−^^1^)
$\vec{v}_{q}$: Velocity of *q*th fluid (m s^−^^1^)
*v*_*t*_ = *μ*_*t*_/*ρ*: Kinematic turbulent eddy viscosity (m^2^ s^−^^1^)
*x*, *y*, *z*: Co-ordinate directions (m)
*Y*_*k*_ and *Y *_*ω*_: Terms representing the dissipation of *k* and *ω* due to turbulence (kg m^−^^1^ s^−^^3^), (kg m^−^^3^ s^−^^2^),
*Y*^+^: *yu*^***^/*v* Where* y* is the depth at which velocity is measured (−)
*α*^*^: Factor used to damp the turbulent viscosity resulting in a Low-Reynolds number correction (−)
*α*_*q*_: The local volume fraction of the *q*th fluid (−)
*α*_*pq*_: The local volume fraction of the *q*th fluid (−)
*α*_*w*_: Local volume fraction of water (−)
*β*: Momentum correction factor (−)
Γ_*ϕ, eff*_: The effective diffusion coefficient (Pa s, kg m^−^^1^ s^−^^1^)
*δx*, *δy*, *δz*: Dimension of fluid elements in a control volume in the *x*, *y* and *z*-directions, respectively (m)
*δ*_*ij*_: Kronecker delta (−)
*ε*: Turbulence dissipation rate (m^2^ s^−^^3^)
*ε*_*ij*_: Dissipation (m^2^ s^−^^3^)
*η*: Kolmogorov length scale (m)
ϑ: Velocity scale (m s^−^^1^)
*μ*: Dynamic viscosity (Pa s, kg m^−^^1^ s^−^^1^)
*μ*_*t*_: Dynamic turbulent viscosity (Pa s, kg m^−^^1^ s^−^^1^)
*μ*_*q*_: Dynamic turbulent viscosity (Pa s, kg m^−^^1^ s^−^^1^)
*ρ*: Density of the fluid (kg m^−^^3^)
*ρ*_*q*_: Density of the *q*th fluid (kg m^−^^3^)
*σ*_*k*_, *σ*_*ω*_: Turbulent Prandtl number for *k* and *ω* respectively (−)
*σ*_*k,1*_, *σ*_*k,2*_, *σ*_*ω,1*_,*σ*_*ω,2,*_* a*_1_: Constants for the *k*–*ω* model (−)
*τ*: Average wall shear stresses (kg m s^−^^2^)
*ϕ*: Represents variables e.g. velocity, pressure
*ϕ*_*ij*_: Pressure strain (kg m s^−^^3^)
*Φ*_*α*_: Local volume fraction (−)
*ω*: Specific turbulence dissipation rate (s^−^^1^)
*ω*_*k*_: Angular velocity (rad s^−^^1^)
$\overline{{\Omega_{ij} }}$: Mean rate of rotation tensor in moving frame (s^−^^1^)
